# Intradermal DNA Electroporation Induces Cellular and Humoral Immune Response and Confers Protection against HER2/neu Tumor

**DOI:** 10.1155/2015/159145

**Published:** 2015-07-13

**Authors:** Alessia Lamolinara, Lorenzo Stramucci, Albana Hysi, Manuela Iezzi, Cristina Marchini, Marianna Mariotti, Augusto Amici, Claudia Curcio

**Affiliations:** ^1^Aging Research Center, G. d'Annunzio University, 66100 Chieti, Italy; ^2^Department of Medicine, Marlene and Stewart Greenebaum Cancer Center, University of Maryland School of Medicine, Baltimore, MD 21201, USA; ^3^Department of Bioscience and Biotechnology, University of Camerino, Camerino, 63100 Macerata, Italy

## Abstract

Skin represents an attractive target for DNA vaccine delivery because of its natural richness in APCs, whose targeting may potentiate the effect of vaccination. Nevertheless, intramuscular electroporation is the most common delivery method for ECTM vaccination. In this study we assessed whether intradermal administration could deliver the vaccine into different cell types and we analyzed the evolution of tissue infiltrate elicited by the vaccination protocol. Intradermal electroporation (EP) vaccination resulted in transfection of different skin layers, as well as mononuclear cells. Additionally, we observed a marked recruitment of reactive infiltrates mainly 6–24 hours after treatment and inflammatory cells included CD11c^+^. Moreover, we tested the efficacy of intradermal vaccination against Her2/neu antigen in cellular and humoral response induction and consequent protection from a Her2/neu tumor challenge in Her2/neu nontolerant and tolerant mice. A significant delay in transplantable tumor onset was observed in both BALB/c (*p* ≤ 0,0003) and BALB-neuT mice (*p* = 0,003). Moreover, BALB-neuT mice displayed slow tumor growth as compared to control group (*p* < 0,0016). In addition, while in vivo cytotoxic response was observed only in BALB/c mice, a significant antibody response was achieved in both mouse models. Our results identify intradermal EP vaccination as a promising method for delivering Her2/neu DNA vaccine.

## 1. Introduction

DNA vaccination is an attractive immunotherapeutic strategy that triggers physiologic immunity and is able to induce long lasting T cell and antibody-mediated tumor protection [1]. In fact, direct injection into mouse muscle or skin of plasmid DNA encoding a selected antigen results in the expression of the gene product and can elicit an immune response against the antigen of interest [2]. Currently, various delivery devices such as gene-guns, bioinjectors, and electroporation systems are being used in order to increase the potency of DNA vaccines [3]. In vivo DNA electroporation (EP) has emerged as an efficient delivery method that allows efficient DNA uptake, long-term and high-level antigen expression [4]. Furthermore, EP is also able to evoke the production of several cytokines and chemokines, thereby increasing the potency of DNA vaccines [4].

Muscle is the most commonly targeted tissue for evaluation of EP in combination with DNA delivery [5]. Under the influence of an electrical field, cellular membranes build up a transmembrane potential until the dielectric strength of the membrane is exceeded and permeation event occurs [6]. Intramuscular electroporation has been previously shown to induce target antigen expression and to trigger humoral and cellular immunity, thus enhancing tumor protection [7, 8]. EP has been used clinically to deliver chemotherapeutic agents to tumor cells in cutaneous malignancies [9, 10]. Nowadays, there are approximately 85 clinical trials listed using electroporation (http://www.clinicaltrials.gov/): around 28 are related to drug delivery and the rest are related to DNA delivery [11–13]. Moreover, several data establish EP as a potent method for stimulating immune responses induced by DNA vaccination in humans [14, 15].

Skin is an attractive site for electroporation in translational settings, as it is readily accessible and EP is minimally invasive and generally well tolerated [16], as compared to muscle. Moreover, skin naturally harbors a high number of antigen presenting cells (APCs), such as Langerhans cells and other types of dermal dendritic cells, which after DNA/antigen uptake can migrate to lymph nodes where efficient presentation to T cells occurs [17, 18], thereby potentially increasing EP efficacy.

The efficacy of intramuscular injection of a plasmid coding for the extracellular and transmembrane domains of the protein product of the Her-2/neu oncogene (ECTM) followed by EP in transgenic murine models has been previously demonstrated [10]. The vaccination protocol induced production of antibodies against Her-2/neu and IFN-*γ* secretion: these two immune activities were associated with the clearance of Her-2/neu spontaneous lesions in transgenic BALB-neuT mice [10]. However, sequential courses of DNA intramuscular EP were required to maintain specific antibody response and counteract the progression of preneoplastic lesions to invasive carcinoma.

In the current study, we evaluated the effectiveness of intradermal vaccination using EP against transplantable Her2/neu^+^ tumor. To address this point, first we analyzed intradermal EP vaccination-induced immune cell recruitment, in both the skin and draining lymph nodes. Second, we evaluated, in both Her2/neu tolerant (BALB-neuT) and nontolerant (BALB/c) mice, the ability of intradermal ECTM EP vaccination to trigger Her2/neu specific immune responses and counteract tumor onset and growth.

## 2. Material and Methods

### 2.1. Mice

Seven-week-old virgin female BALB/c and BALB-neuT mice (H-2^d^) were used. BALB/c mice were from Charles River Laboratories (Calco, Italy). Virgin BALB-neuT mice, overexpressing the transforming rat Her-2/neu oncogene under the control of the mouse mammary tumor virus [19], were bred in house. Mice of the same age were randomly assigned to experimental groups and were treated according to the European Community guidelines.

### 2.2. Injection of Plasmids or FITC-Dextran and Electroporation

pVAX (Invitrogen, Milan, Italy) was the backbone for all the vaccines. The cDNA sequence for ECTM was obtained as previously described [19]. The pVAX-EGFP DNA construct was obtained by subcloning the EGFP cDNA, excised from pEGFP-N1 (Clontech, Mountain View, CA) by HindIII/XbaI digestion, into the HindIII/XbaI sites of the pVAX-1 vector (Life Technologies, Carlsbad, CA). The inserted sequence was verified by sequencing.

All plasmids for DNA immunizations were grown in* E. coli* DH5*α* strain, and large-scale preparation of the endotoxin-free plasmid DNA was carried out using Qiagen EndoFree Plasmid-Giga kits (Qiagen, Chatsworth, CA, USA) according to the manufacturer's instructions. Endotoxin-free plastic ware was used to prevent recontamination of the plasmid.

Anesthetized mice were vaccinated with 50 *μ*L of solution containing 50 *μ*g of DNA or 25 mg/mL of fluorescein isothiocyanate-dextran (FITC-dextran) average mol wt 40,000 (Sigma Aldrich, Milan, Italy). The vaccination course consisted of two intradermal injections into the skin of the back near the base of the tail. Immediately after the plasmid or FITC-dextran administration, a conducting gel and an electrode were placed over the injection site and voltage was set up according to previously described protocols (2 pulses, 1125 V/cm 50 *μ*s, and 8 pulses, 275 V/cm 10 msec) [20]. Electrodes conslehisted of two parallel lamellae at 6–8 mm distance. Pulses were generated by an Igea Cliniporator (Igea, Carpi, Italy). BALB/c and BALB-neuT mice were vaccinated 21 and 7 days before a subcutaneous injection with a lethal dose of TUBO cells (day 0).

### 2.3. Cell Lines

Her-2/neu positive TUBO cells had been originally isolated from a carcinoma arising in a BALB-neuT mouse [21]. N202.1A (Her-2/neu positive) and N202.1E (Her-2/neu negative) lines had been isolated from a mammary carcinoma in a FVB/N mouse (H-2^q^) transgenic for the rat Her-2/neu protoncogene [22]. TUBO cells were cultured in DMEM (BioWhittaker Europe, Verviers, Belgium) with 20% FBS (Life Technologies, Inc., San Giuliano, Italy), and N202.1A and N2021E in RPMI (BioWhittaker Europe) with 10% FBS (Life Technologies).

### 2.4. Tumor Challenge

Mice were subcutaneously injected with 0.2 mL of a single cell suspension containing the minimal lethal dose of TUBO (10^5^) cells [19] in the right flank. Mice were checked twice weekly for tumor onset: stable or growing masses were regarded as tumors. Tumor growth was evaluated twice weekly in a blind fashion. Neoplastic masses were measured with calipers in the two perpendicular diameters, and the tumor volume was recorded for 150 days. At the end of this period, tumor-free mice were classified as survivors. Mice were euthanized when the tumor exceeded 1 cm^3^ volume for humane reasons.

### 2.5. Antibody Response

Sera were collected 14, 30, 80, and 100 days after vaccination. 100 *μ*L of 1 : 10 diluted sera was incubated for 45 minutes at 4°C with 2 × 10^5^ N2021A or 1E cells pretreated with Fc receptor blocker (CD16/CD32; Pharmingen, Milan, Italy) for 15 minutes at 4°C. Total Ig binding was evaluated using a PE-conjugated goat anti-mouse Ig antibody (DakoCytomation, Milan, Italy). The Ab4 (Oncogene, Milan, Italy) was used as positive control for anti-Her2/neu reactivity. Results were expressed as mean fluorescence intensity (MFI) [23]. N202.1E MFI was subtracted to N202.1A MFI to identify Her2/neu specific antibody reactivity and differences in MFI were analyzed by Student's *t*-test.

Isotype determination was carried out by an indirect immunofluorescence procedure. Dilutions (1 : 10) of sera in PBS-azide-BSA were incubated for 45 minutes at 4°C with 2 × 10^5^ N202.1A or N202.1E cells, pretreated with Fc receptor blocker (CD16/CD32; Pharmingen) for 15 minutes at 4°C. After washing, the cells were incubated for 30 minutes with rat Alexa 488-conjugated antibodies anti-mouse IgM, IgG1, IgG2a, IgG2b, or IgG3 (Invitrogen) and fluorescence evaluated. The specific N202.1A-binding potential was calculated as the percentage of MFI/total Ig.

Flow cytometry experiments were performed using a FACSCalibur (Becton Dickinson, Milan, Italy) and analyzed with FlowJo software (FlowJo LLC, Ashland, USA).

### 2.6. In Vivo Cytotoxicity Assay

In vivo cytotoxicity assay was performed as previously described [24], with slight modifications. Briefly, a single-cell suspension of 10^7^ naive splenocytes (Spc)/mL was labeled with 0.5 (CFSE^low^) or 5 *μ*mol/L (CFSE^high^) of the carboxyfluorescein diacetate succinimidyl ester CFSE (Molecular Probes, Leiden, Netherlands). Spc labeled with 5 *μ*mol/L CFSE were also pulsed with 15 *μ*g/mL of H-2K dominant Her2/neu (TYVPANASL) (Inbios SRL, Naples, Italy) peptide for 1 hour at room temperature. The two Spc populations were mixed together in equal amounts and injected i.v. into control and treated mice. Mice were sacrificed 48 hours later, and single-cell suspensions from spleens were processed individually to evaluate the presence of CFSE^high^ cells with the FACSCalibur after adding propidium iodide to exclude dead cells. The specific cytolytic activity was calculated as 100 × (percentage CFSE^low^ cells − percentage CFSE^high^ cells)/percentage CFSE^low^ cells.

### 2.7. Immunohistochemistry and Immunofluorescence

For immunohistochemical evaluation, mice were euthanized at 3, 6, 24, 48, 72, and 96 hours after vaccination and the skin and axillary, inguinal, and popliteal lymph nodes were collected, fixed in PFA 1%, and embedded in OCT and tissue sections were stained with hematoxylin and eosin (H&E) for histological examination. Skin and lymph nodes sections were incubated with the following primary antibodies: rat mAb anti-CD4, anti-CD8*α*, anti-CD11b, anti-CD45R/B220, anti-Gr-1 (BD Pharmingen, Milan, Italy), anti-CD68 (Abcam, Cambridge, UK), and anti-CD11c (Chemicon International, USA). After washing, slides were overlaid with appropriate secondary antibodies. Immunostaining was developed with Vulcan Fast Red (Biocare, Milan, Italy) alkaline phosphatase method. For immunofluorescence, secondary antibodies conjugated with Alexa 488 and Alexa 546 (Invitrogen, Life Technologies, Monza, Italy) were used. Nuclei were stained with DRAQ5 (Alexis, Life Technologies, Monza, Italy), YO PRO-3, or TO PRO-3 (Molecular Probes, Monza, Italy). Image acquisition was performed using Zeiss LSM 510 Meta confocal microscope (Carl Zeiss SpA, Milan Italy).

The slides were examined in a double-blind fashion, and digital images of representative areas were taken.

### 2.8. Statistics

Quantitative data are presented as mean ± SEM from three independent experiments. The significance of differences was evaluated with two-tailed Student's *t*-test or log-rank (Mantel-Cox) test. Statistical analysis was carried out with GraphPad Prism5 Software (San Diego, CA, USA); *p* ≤ 0.05 was used as the critical level of significance.

## 3. Results

### 3.1. Intradermal EP Vaccination Results in a Marked Recruitment of Reactive Infiltrates

**Figure 1:**
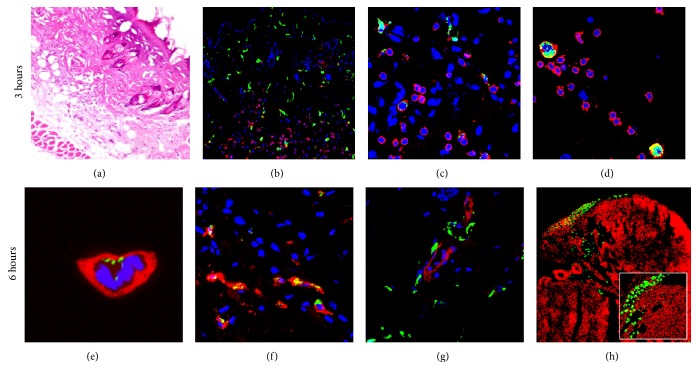
Electroporation effect on FITC-dextran expression and distribution in skin. Three hours after intradermal injection of FITC-dextran followed by EP reactive cells that are recruited (a). Among these CD11b (red) cells incorporate FITC-dextran and are found in different skin layers ((b), (c), (d)). Six hours after vaccination, CD11c (red (e)) Lyve 1 (lymphatic vessel, red (f)) and CD31 (endothelial vessels, red (g)) cells are detected in the skin. FITC-dextran and nuclei are shown in green and blue, respectively ((b)–(g)). FITC-dextran incorporating cells are observed in draining lymph nodes 6 hours after treatment (h). Nuclei are shown in red (h). Images are representative of three independent experiments. Magnification: (a), (b) ×200; (c), (d), (f), (g) ×630; (e) ×1500; (h) ×200, insert ×400.

To analyze the cellular effects of vaccination, intradermal injection of FITC-dextran followed by EP was performed in BALB/c mice. Immediately after vaccination (3 hours) skin showed tissue damage and a coagulated aspect as well as evident inflammatory infiltrate (Figure 1(a)). Immunofluorescence analysis revealed the presence of CD11b macrophages with incorporated FITC-dextran (Figures 1(b)–1(d)) in close proximity of the damaged area as well as in epidermis (Figure 1(b)), dermis (Figure 1(c)), and hypodermis (Figure 1(d)). At this extremely early time point, no cells with encapsulated FITC-dextran were detected in lymph nodes (not shown). At a later time point (6 hours after vaccination), CD11c cells with incorporated FITC-dextran were detected in the dermis (Figure 1(e)). Additionally, FITC-dextran was also incorporated by Lyve 1 lymphatic endothelial cells (Figure 1(f)) and deposited outside CD31 blood vessels (Figure 1(g)). Some cells with incorporated FITC-dextran were found into inguinal lymph nodes (draining lymph nodes) (Figure 1(h)). No FITC-dextran incorporating cells were detected in control mice intradermally injected without electroporation. Altogether these data are consistent with an efficient transfection of APCs and their progressive migration through the skin layers to lymph nodes.

**Figure 2:**
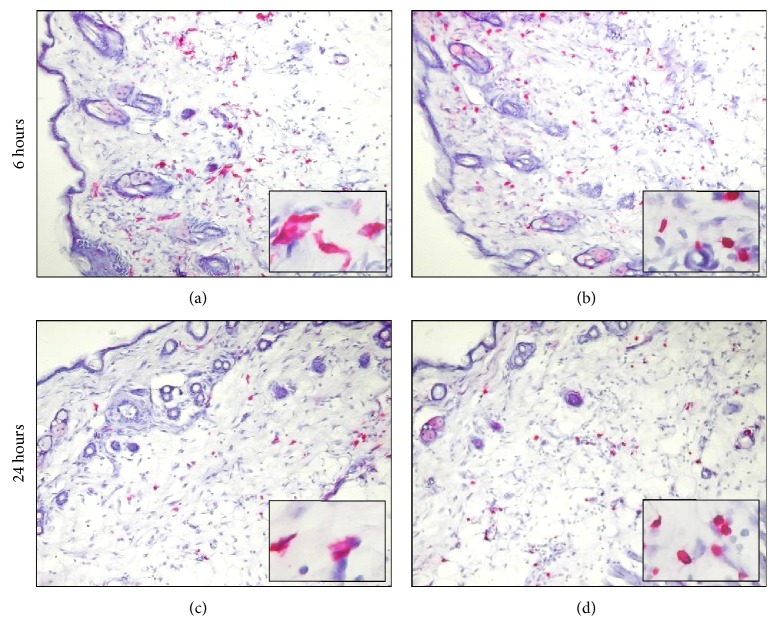
APCs infiltrate vaccinated skin. Six hours after intradermal EP vaccination dendritic (a) and CD4 (b) cells infiltrate the electroporated skin and decrease starting at 24 hours after treatment ((c), (d)). Red staining shows positivity for CD11 ((a), (c)) or CD4 ((b), (d)) molecule. Magnification: ×200, insets: ×400. Images are representative of three independent experiments.

Accordingly, immunohistochemistry analysis highlighted the accumulation of granulocytes (Gr1) and macrophages (CD11b) 3 hours after treatment (Table 1) and a significant number of reactive cells were still observed at 6 hours. In particular, at this time point, dendritic cells (CD11c) and T lymphocytes (CD4) were detected (Table 1 and Figures 2(a) and 2(b)). Twenty-four hours after vaccination the number of dendritic and CD4 cells decreased until almost baseline levels (Table 1 and Figures 2(c) and 2(d)) and the local inflammatory infiltrate gradually turned off starting at 48 hours after vaccination.

**Figure 3:**
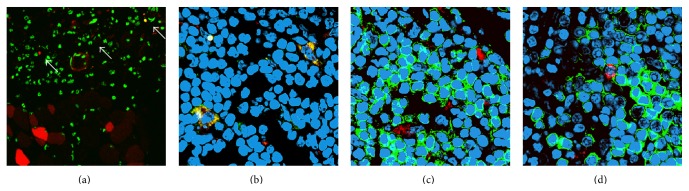
Intradermal EP vaccination transfects dendritic cells that migrate to draining lymph nodes. Transfected (red) muscle fibers and cells (arrows) in skin. Nuclei are shown in green. Magnification ×400. (a) Transfected dendritic cells have migrated into lymph nodes (CD11c in green, EGFP in red). (b) Interaction of dendritic cells that have incorporated EGFP (in red) with CD4 cells (in green) (c). Transfected cells (in red) adjacent to B220 cells (in green) in draining lymph node. (d) Nuclei are shown in blue, magnification ×630. ((b)–(d)) Images are representative of three independent experiments.

In order to shed light into the mechanisms underlying the immune responses elicited by the intradermal vaccination, we analyzed the type and the localization of pVax EGFP transfected cells in both skin and inguinal draining lymph nodes (Figure 3). After an intradermal injection probably the major part of the volume is deposited in the dermis due to the epidermis being very thin, particularly in mice. However, twenty-four hours after vaccination, positive cells have also been found in epidermis and in the subcutaneous muscle layer, the panniculus carnosus, near the damaged zone (Figure 3(a)). Close to them, some infiltrating cells expressed the transgene too (Figure 3(a)). As shown in Table 1, dendritic cells decreased in skin 24 hours after treatment, so we investigated the presence of these cells in draining lymph nodes. Not only transfected dendritic cells were found (Figure 3(b)), but also, in accordance with their APC role, these cells were transfected and in very tight proximity with both CD4 (Figure 3(c)) and B220 (Figure 3(d)).

### 3.2. Intradermal EP Vaccination Protects against a Lethal Tumor Challenge by Inducing Her2/neu Specific CTLs and Antibody Response

**Figure 4:**
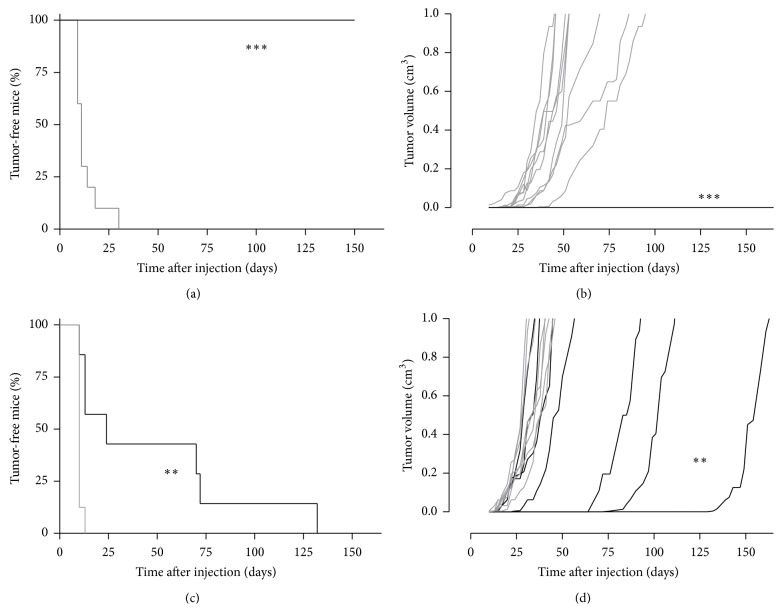
Intradermal EP vaccination protects BALB/c and BALB-neuT mice against a TUBO challenge. Mice were immunized twice with empty (pVAX, gray line) or ECTM (pVAX-ECTM, black line) plasmid. pVAX-ECTM treated BALB/c mice (*n* = 12) display total tumor protection ((a), *p* < 0,0001), while control group (*n* = 10) develops fast growing tumors within 30 days after challenge ((b), *p* = 0,0003). Vaccinated BALB-neuT mice (*n* = 7) display a delayed tumor onset ((c), *p* = 0,0030) and slower tumor growth (*p* < 0,0016) with respect to control group (*n* = 8). Each line refers to an individual tumor. Log-rank (Mantel-Cox) test and Student's *t*-test were used for statistical analysis.

Intradermal ECTM EP vaccination effectively delayed tumor onset in both Her2/neu nontolerant and tolerant mice. In fact, while mice vaccinated with an empty plasmid developed tumor within 30 days (Figures 4(a) and 4(c)), in pVAX-ECTM treated group, all BALB/c mice remained tumor free until the endpoint (Figures 4(a) and 4(b)), and BALB-neuT mice displayed a strong delay in tumor onset (*p* = 0,003) (Figure 4(c)).

Notably, tumor growth in these mice was characterized by very slow kinetics as compared to pVAX group (*p* < 0,0016) (Figure 4(d)).

**Figure 5:**
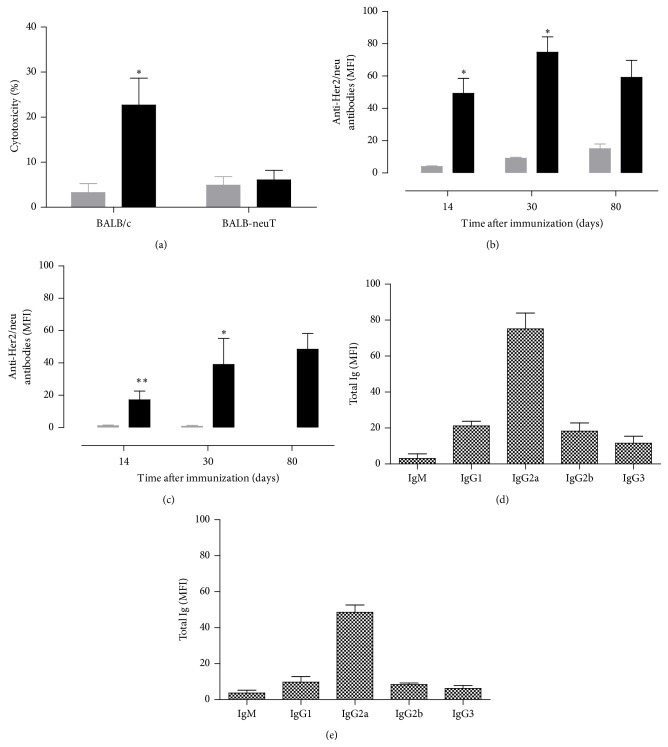
Intradermal EP vaccination induces cellular response in Her2/neu nontolerant mice and humoral immune response in both tolerant and nontolerant mice. In vivo cytotoxicity against the H-2^d^ dominant peptide in BALB/c and BALB-neuT (each experimental group *n* = 3) mice vaccinated with pVAX (gray bars) or pVAX-ECTM plasmid (black bars). Data are reported as % of lysis. ^*∗*^ *p* = 0,0369, Student's *t*-test. (a) Anti-Her2/neu antibody titer (MFI ± SEM) in BALB/c (b) and BALB-neuT mice (c). ^*∗∗*^ *p* = 0,0096, ^*∗*^ *p* < 0,0242, Student's *t*-test ((b), (c)). Isotype titration, performed in three different pools of sera, revealed similar composition of Her2/neu specific antibodies for BALB/c (d) and BALB-neuT (e) treated mice ((d), (e)).

In accordance with the observed total protection against tumor challenge, higher CTL activity was observed in BALB/c mice electroporated with pVAX-ECTM plasmid (*p* = 0,0369), while no cytotoxicity was detected in Her2/neu tolerant BALB-neuT mice (Figure 5(a)). This data together with a higher protection observed in Balb-c mice is consistent with a higher immune tolerance of BALB-neuT mice.

Moreover, 14, 30, and 80 days after last vaccination, the titer of anti-Her2/neu antibody was evaluated. A significant Her2/neu specific antibody titer was detected in both BALB/c (*p* = 0,0256) and BALB-neuT (*p* = 0,0096) pVAX-ECTM vaccinated mice as compared to pVAX groups (Figures 5(b) and 5(c)) already after 14 days. Interestingly the titer remained elevated also 30 days after the vaccination (BALB/c *p* = 0,0242, BALB-neuT *p* = 0,0197), with detectable high levels up to 100 days after vaccination (not shown) in all surviving mice. The induction and persistence of the humoral response in both mouse models, together with the similar isotype profiling of anti-Her2/neu reactive antibodies (Figures 5(d) and 5(e)), indicate that this type of immunity is similarly triggered in both tolerant and nontolerant mice. Interestingly a high antibody titer correlated with tumor protection in BALB-neuT mice (see Supplementary Figure 1 in Supplementary Material available online at http://dx.doi.org/10.1155/2015/159145). It is important to note that naked DNA injection resulted in negligible antibody or cytotoxicity induction and tumor protection (Supplementary Figure 2).

Altogether these results indicate that our vaccination protocol is able to induce a protective immune response not only in Her-2 nontolerant BALB/c mice, but also in tolerant BALB-neuT mice.

## 4. Discussion

By virtue of its versatility and efficacy in inducing immunity against selected antigens, DNA vaccination represents a suitable approach and may provide new therapeutic avenues for the treatment of tumors [25]. Plasmids can be delivered by intramuscular, intradermal/epidermal, subcutaneous, oral (e.g., with bacterial carrier), and pulmonary (e.g., aerosols) or other routes (e.g., vaginal) [26]. Notably, the skin is a particularly attractive site for vaccination given that intradermal vaccination is easy to perform and well tolerated both clinically and histopathologically [17]. Additionally, skin is characterized by an extended local network of dendritic cells and the easy access to the skin-draining lymph nodes generates effector T cells and immunoglobulin-producing B cells [27, 28]. These characteristics may help the generation of a long-term protective immunity [29, 30]. In fact, experiments with bone marrow chimeras have shown that APCs, presumably dendritic cells, have a key role in DNA vaccination-induced protection [26]. Moreover, it is already known that using intradermal EP vaccination a wide variety of cell types can be transfected and these cells can be found in the draining afferent lymph nodes, suggesting that migration of directly transfected dendritic cells may occur [17]. Nevertheless, intradermal vaccination is notorious for its lower antibody responses compared to intramuscular route of plasmid administration in mice [31–34].

In this study we demonstrate that intradermal in vivo application of pulsed electric field confers long-term protection from Her2/neu tumor. Indeed, we report that efficiently permeabilized cells are found in all layers of the skin and migrate to draining lymph nodes. This is consistent with previous studies showing that, in contrast to the predominantly epidermal injection of naked DNA [35], a pattern of dermal and subdermal transfection is observed in the skin [10]. Concordantly with our data (Figures 1 and 2) transfected cell types included numerous mononuclear cells with dendritic morphology, as well as large numbers of adipocytes [17]. The variety of transfected cell types is likely to grant the mounting of an effective and durable immune response: in fact it has been demonstrated that transfection of different cell types results into different immune responses [36–39].

We further demonstrated that CD11c are efficiently permeabilized and hopefully transfected upon DNA vaccination (Figure 3 and Supplementary Figure 2) as a result of electroporation. The preferential EP mediated transfection of cells with dendritic morphology could provide a distinct advantage due to the central role of such cells in the stimulation of both primary and secondary immune responses [17]. Dendritic cells are the key initiators and regulators of any immune response which determine the outcome of CD4 and CD8 T cell responses [30]. Nevertheless, given the fact that either direct transfection of APCs or uptake of protein from other transfected cells (cross presentation) [26] may occur, we cannot provide formal proof that, although efficiently permeabilized, skin APCs are directly transfected. However, either pathway may lead to an effective protection. In fact it has been demonstrated that, in response to vaccination, the cutaneous immune system sets up complex mechanisms including (1) recognition and capture of the vaccine by skin-resident APCs or by recruited innate immune cells (inflammatory microenvironment), (2) activation of skin APCs, (3) passive diffusion or cell transport of antigens to the secondary lymphoid organs draining the skin (draining lymph nodes), (4) processing and presentation of the antigens to immature CD4 and CD8 T cells by activated APCs or to naive follicular B cells, (5) activation of antigen-specific CD4 and CD8 T cells and clonal expansion as well as activation of specific B cells within a germinative center, (6) migration of specific effector CD4 and CD8 T and B cells toward the zone of vaccination and elimination of the vaccine antigens, (7) generation of a pool of specific memory T and B cells in the secondary lymphoid organs and in the periphery (skin and mucosa) [30].

Indeed, in our study, after intradermal EP vaccination, a significant number of reactive cells were already observed at 6 hours after treatment, consisting primarily of dendritic cells.

Independent on the mechanism by which APCs acquire and then present the target antigen, to the best of our knowledge, this is the first report showing that intradermal DNA injection targeting Her2/neu followed by EP elicts a strong inflammatory-like response and induces both CTL and antibody responses in BALB/c and humoral response in tolerant BALB-neuT mice. This is consistent with recent reports of the presence of both CTL and antibody responses after vaccination in other tumor models [40, 41]. A previous study suggested that intradermal plasmid injection could not induce an effective immune response in Her2/neu models [25]. Nevertheless, our study demonstrates that, similar to what have been reported for intramuscular route, electroporation boosts the efficacy of ECTM vaccination when intradermally administered (Supplementary Figure 1), resulting in a specific immunity and tumor protection. Additionally, previous studies demonstrated that, in BALB-neuT mice, intramuscular ECTM injection followed by EP repeated every 10 weeks triggered a humoral response that inhibited the progression of spontaneous mammary carcinogenesis [10]. Although we cannot directly compare the two methods, in our study, a single cycle of intradermal EP vaccination induced a significant delay in tumor onset with an antibody titre that remains high over time, indicating intradermal route as a valid alternative to induce Her2/neu specific response. This data confirms previous studies implicating anti-Her2/neu antibodies in the inhibition of Her2/neu-driven carcinogenesis and progression [7, 22, 42, 43]. The importance of antibodies is further underlined by the reported absence of protection in BALB-neuT/*μ*KO mice unable to produce antibodies to Her2/neu after intramuscular DNA vaccinations with ECTM plasmid [30]. Moreover, the high titer of anti-Her2/neu antibodies has been demonstrated to correlate with the downregulation of Her2/neu in the cells of mammary lesions and its cytoplasmic confinement [21, 43, 44]. Consistent with a central role of antibody-mediated protection [45–48] we observed protection from tumor challenge in BALB-neuT mice even in the absence of cell cytotoxicity, and long-term survivor mice displayed the highest antibody titer (Supplementary Figure 2).

Notably in our study we were able to achieve a long-term protection not only in Her2/neu nontolerant BALB/c mice that are more prone to mount an effective immune response against the oncogene (as demonstrated also by the capability of our vaccination to induce cytotoxicity in these mice), but also in highly tolerant BALB-neuT mice. BALB-neuT mice are genetically predestined to develop lethal invasive carcinomas in their mammary glands by week 22 [10]. As the transforming (and immunization target) oncogene is embedded in the genome of these mice, a unique dynamic relationship between the oncogenic signals and inhibitory immune reactions is taking place. Given the long-term protection achieved with only 2 vaccination rounds, it will be of interest to test our experimental setting on spontaneous Her2/neu positive mammary tumors to evaluate if intradermal EP vaccination can reduce the number of doses and/or prolong the tumor-free mice. In fact, prolongation of immune memory could be even a more worthwhile goal than enhancement of the immune response [42]. Indeed, activating the appropriate arm of the immune system that induces protection is now one of the major challenges to be faced in vaccine development against tumors. However, further studies are necessary to find out the best settings for vaccination, such as the nature of the antigens, against which immune responses are elicited, the use of adjuvant, and specific delivery system that will ensure optimal presentation to immune system and protection from tumors.

## Supplementary Material

Supplementary Material Figure 1: Sera were collected 38 days after vaccination and Her2/neu specific antibody reactivity was evaluated as described in material and method section of the manuscript. The antibody titer was compared to times of first palpable tumors for each vaccinated mouse.
Supplementary Material Figure 2: BALB-neuT mice were vaccinated with or without electroporation with pVAX or pVAX-ECTM as described in material and methods section of the manuscript. Seven days after the last vaccination, all mice were subcutaneously challenged with a lethal dose of TUBO cells (10^5^) and tumor growth was evaluated for 80 days. Sera were collected 14, 30 and 80 days after immunizations and analyzed as reported in the manuscript. Skin sections were stained with anti-CD11b and anti-CD11c according to the protocol described in the methods section.

## Figures and Tables

**Table 1:** Semiquantitative analysis of inflammatory cells present in the skin of BALB/c mice from 3 to 96 hours after pVAX-EGFP vaccination followed by electroporation.

Time after intradermal EP vaccination	Gr-1	CD11b	CD68	CD11c	CD4	B220	CD8
3 h	+++	+++	++	+	+	−/+	−
6 h	++	+++	++	++	+++	+	−/+
24 h	++	++	+	+	++	+	−/+
48 h	+	++	+	−/+	+	++	−
72 h	+	++	+	−/+	+	++	−
96 h	−/+	++	+	−/+	−/+	++	−

The expression of reactive cells was defined as absent (−), scarcely (−/+), moderately (+), frequently (++), or strongly (+++) present on cryostat sections stained with the antibodies.
