# Supplementary figures and images for: Intradermal DNA Electroporation Induces Cellular and Humoral Immune Response and Confers Protection against HER2/neu Tumor
Source: J Immunol Res. 2015 Jul 13;2015:159145. doi: 10.1155/2015/159145 (PMC4515534; doi:10.1155/2015/159145)

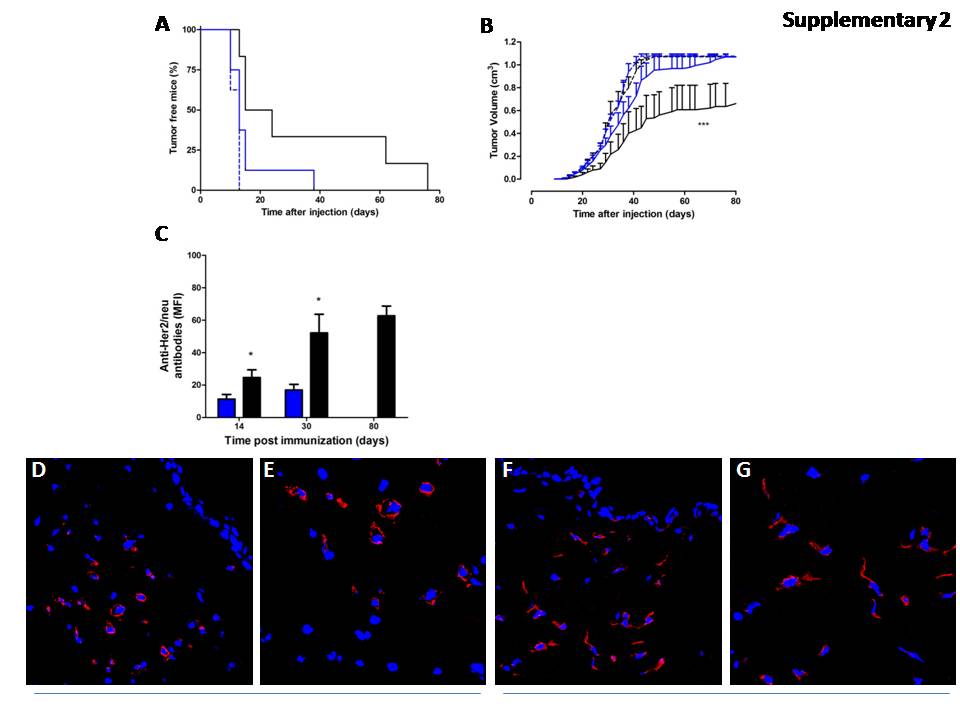

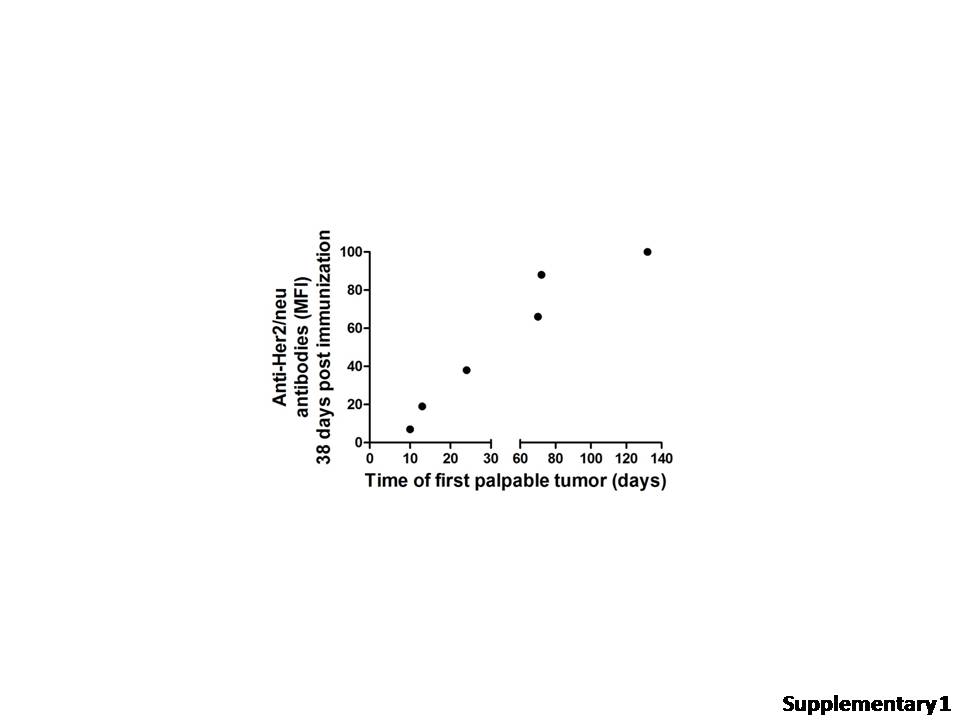

Supplement: Supplementary file 1 — Supplementary Material Figure 1: Sera were collected 38 days after vaccination and Her2/neu specific antibody reactivity was evaluated as described in material and method section of the manuscript. The antibody titer was compared to times of first palpable tumors for each vaccinated mouse. Supplementary Material Figure 2: BALB-neuT mice were vaccinated with or without electroporation with pVAX or pVAX-ECTM as described in material and methods section of the manuscript. Seven days after the last vaccination, all mice were subcutaneously challenged with a lethal dose of TUBO cells (105) and tumor growth was evaluated for 80 days. Sera were collected 14, 30 and 80 days after immunizations and analyzed as reported in the manuscript. Skin sections were stained with anti-CD11b and anti-CD11c according to the protocol described in the methods section. [file 159145.f1.doc]
